# Supplementary material for: The Catalytic-Dependent and -Independent Roles of Lsd1 and Lsd2 Lysine Demethylases in Heterochromatin Formation in Schizosaccharomyces pombe
Source: Cells. 2020 Apr 13;9(4):955. doi: 10.3390/cells9040955 (PMC7226997; doi:10.3390/cells9040955)
Supplement: Supplementary file 1 [file cells-09-00955-s001.tgz › Supplemental figures_Marayati.pdf]

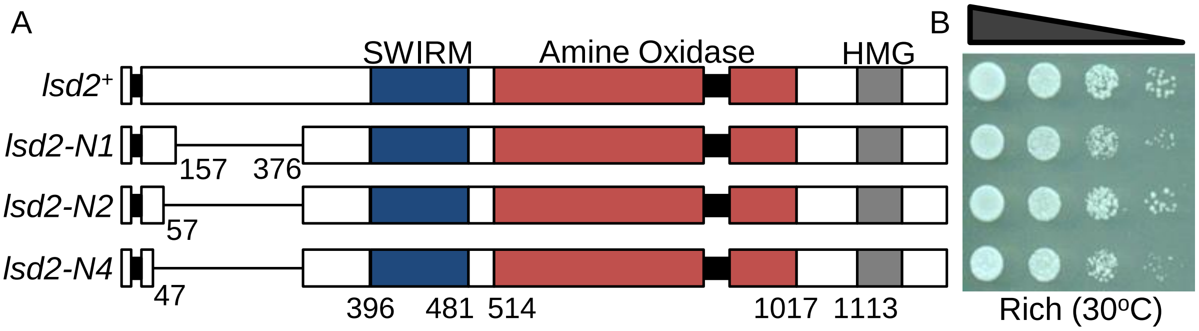

Supplemental Figure S1. Lsd2 N-terminal truncations retaining the nuclear localization sequence (S35-D47) display either no, or mild, growth defects. (A) Schematic representation of N-terminal mutants. Numbers indicate the position in the amino acid sequence of Lsd2. (B) Serial dilution of *lsd2-N1*, *lsd2-N2*, and *lsd2-N4* cells show no significant growth defects on rich medium plates at 30°C.

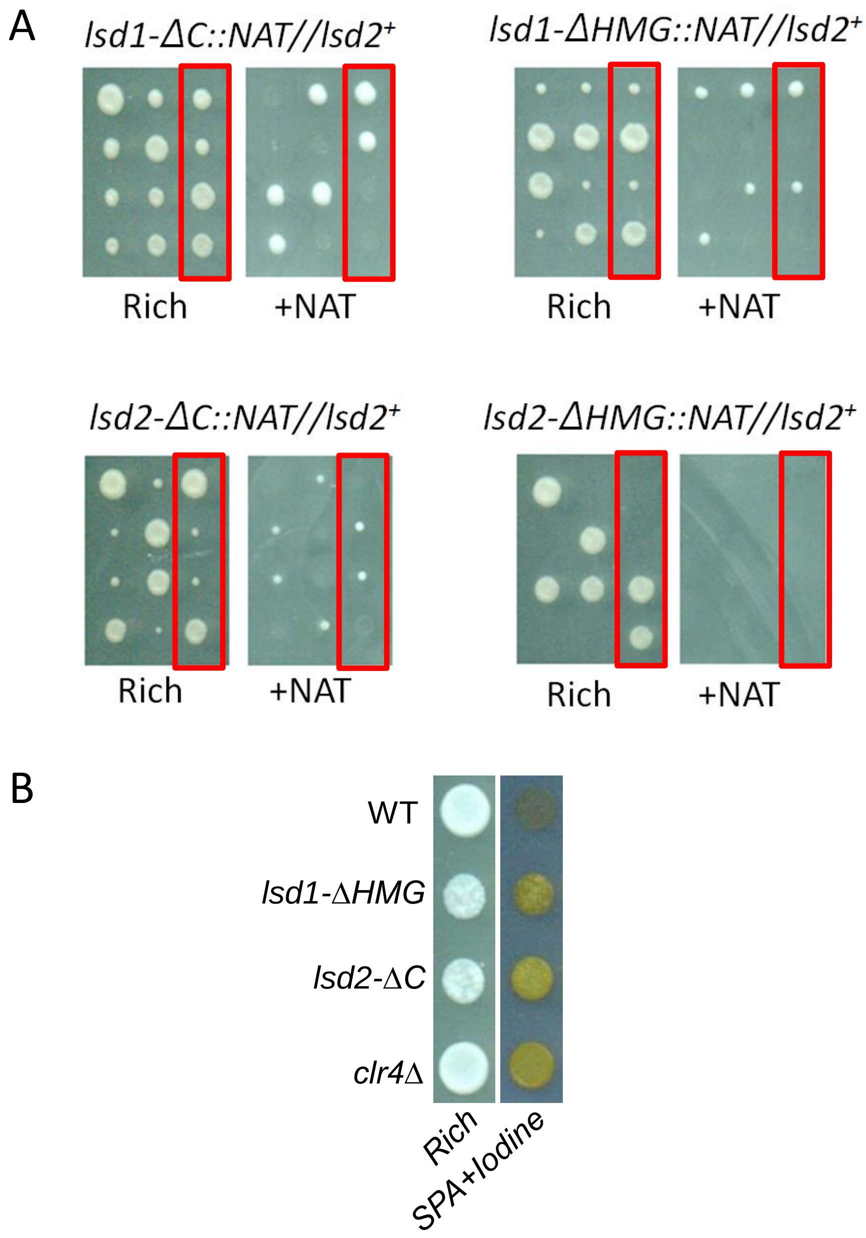

Supplemental Figure S2. *lsd2-ΔHMG* cells are not viable and *lsd1-ΔC*, *lsd1-ΔHMG,* and *lsd2- ΔC* cells have growth defects. (A) Heterozygous diploids containing one copy of the mutant allele (linked with resistance to the antibiotic Nourseothricin (NAT)) and one copy of the WT allele were sporulated and tetrads of spores were arranged in columns and grown to colonies on rich media. Once colonies of sufficient size were formed, they were replica plated on to rich media containing the appropriate antibiotic in order to identify colonies with mutant alleles. Viable *lsd1-ΔC*, *lsd1-ΔHMG,* and *lsd2- ΔC* form colonies on both rich media and antibiotic-containing media. *lsd2-ΔHMG* strains, however, are only observed on rich media without NAT. Red box: the same individual tetrad that grow on rich medium with or without NAT. (B) Cells with indicated genotypes were plated on SPA media to induce sporulation and subsequently exposed to iodine vapor. Cells with proficient sporulation show darker staining patterns than cells with sporulation defects.

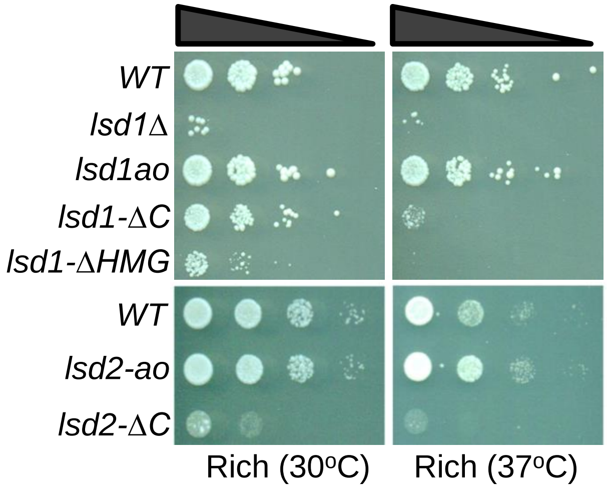

Supplemental Figure S3. Serial dilution demonstrating the relative growth defects and heat sensitivities (37°C) of Lsd1 and Lsd2 mutant strains (schematic of mutations in each indicated strain are displayed in Figure 2A).

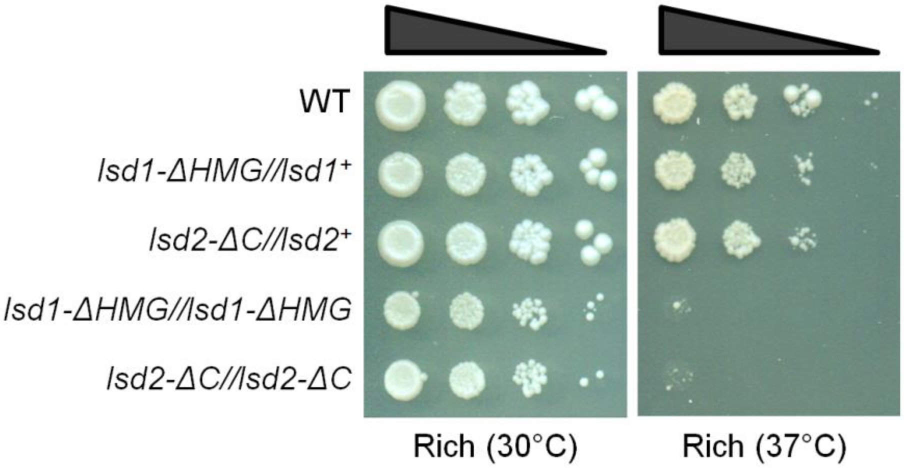

Supplemental Figure S4. Lsd1 and Lsd2 C-terminal mutations do not have dominant negative effects. Serial dilution of heterozygous and homozygous diploid Lsd mutant strains shows that the relative growth defects and heat sensitivities (37°C) of homozygous diploid mutants are rescued in heterozygous diploid strains.

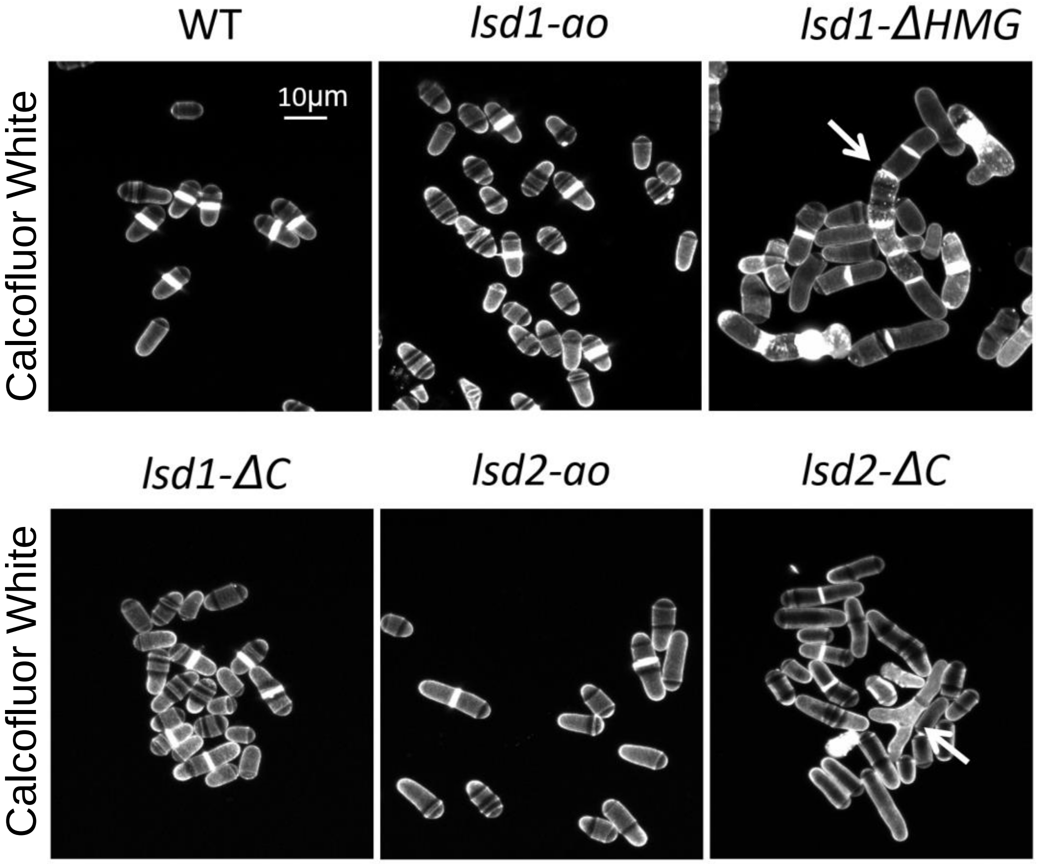

Supplemental Figure S5. The C-terminal domains of Lsd1 and Lsd2 are vital for proper growth and morphology. Calcofluor white staining of cell walls reveals morphological defects in mutant cells. Arrows indicate sample cells with severe morphological aberrations.

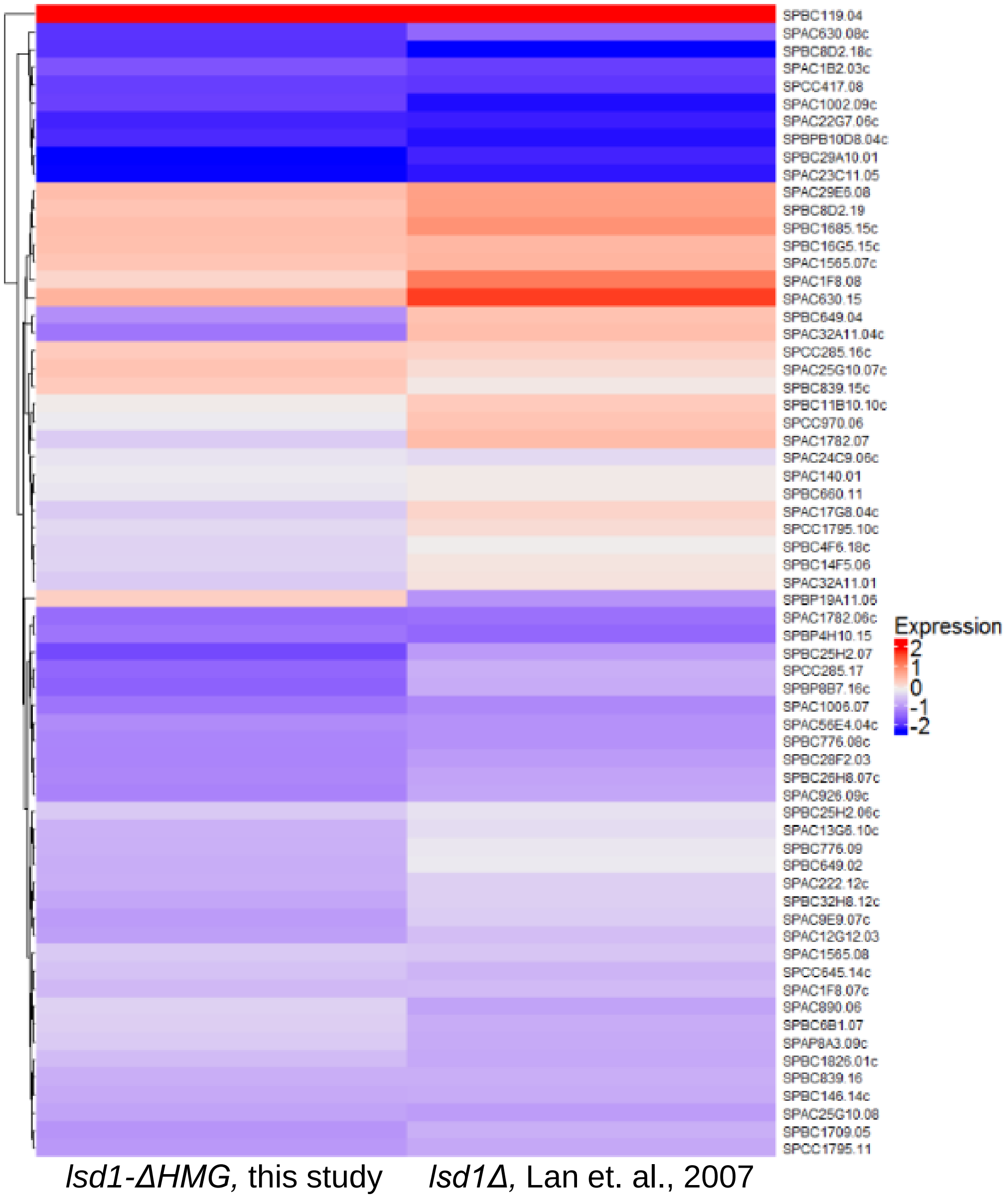

Supplemental Figure S6. The similarity between RNA-seq analysis (*lsd1-ΔHMG,* this study) and expression array data (*lsd1Δ,* Lan et. al., 2007). The expression profiles for the 68 highest-confidence target genes of Lsd1 are shown (see supplemental table S3 for complete list of genes).

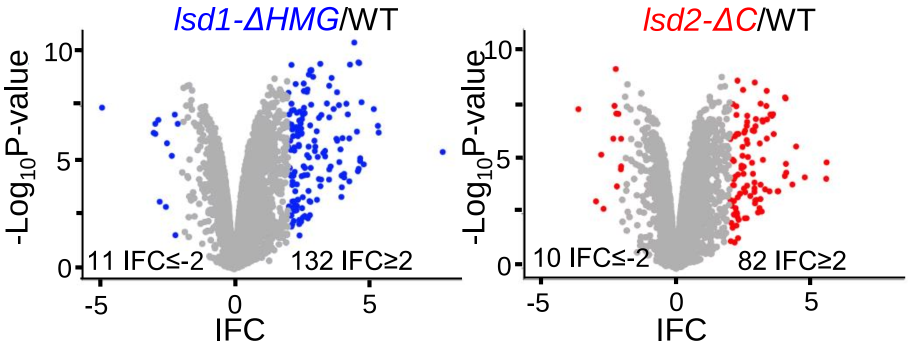

Supplemental Figure S7. Volcano plots highlighting significant genes that are differentially expressed (DEGs) in *lsd1-ΔHMG* or *lsd2-ΔC* mutants using RNA-seq. lFC: Log fold changes.

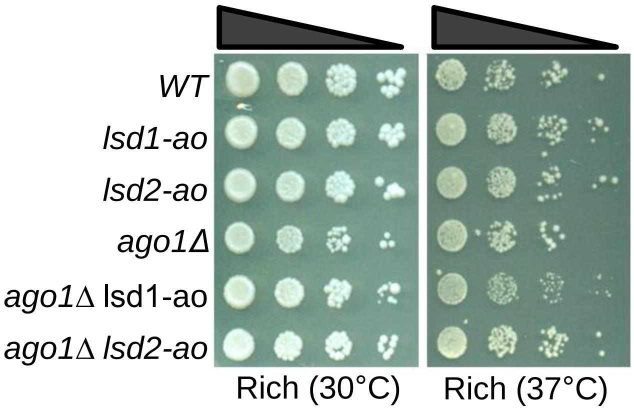

Supplemental Figure S8. Serial dilutions demonstrating the genetic interactions between *lsd1* and *lsd2* catalytic mutant strains combined with *ago1Δ.*

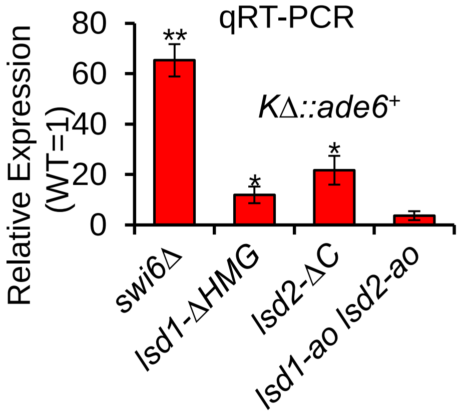

Supplemental Figure S9. *ade6*$^+$ expression from cells collected in Figure 5B was monitored by qRT-PCR. Relative expression to WT is shown. *$p \leq 0.05$ and **$p \leq 0.01$ as determined by student's *t* test comparing the indicated samples with WT for qRT-PCR. Error bars represent *s.e.m.*

Strains	*lsd2-ΔC*	*lsd1-ΔHMG*	*lsd1-ao*	*lsd2-ao*	*ago1Δ*	*epe1Δ*	*clr3Δ*	*sir2Δ*	*clr6-1*
lsd1-ao	**negative**	**lethal**	**n/a**	**positive**	**positive**	**n/a**	**n/a**	**n/a**	**n/a**
lsd2-ao	**lethal**	**lethal**	**positive**	**n/a**	**positive**	**n/a**	**n/a**	**n/a**	**n/a**
lsd1-ΔHMG	**lethal**	**n/a**	**lethal**	**lethal**	**negative**	**positive**	**negative**	**negative**	**positive**
lsd2-ΔC	**n/a**	**lethal**	**negative**	**lethal**	**negative**	**positive**	**negative**	**negative**	**positive**

Supplemental Figure S10. A summary table showing all the genetic interactions between the Lsd1/2 mutants and silencing factors that were investigated in this study. Blue: Negative interactions including lethality; Red: Positive interactions; n/a: data not available.
